# Wide-Awake Local Anesthesia With no Tourniquet Versus General Anesthesia for the Plating of Distal Radius Fracture: A Systematic Review and Meta-Analysis

**DOI:** 10.3389/fsurg.2022.922135

**Published:** 2022-06-27

**Authors:** Ting-Yu Tu, Chih-Yang Hsu, Pei-Chin Lin, Chun-Yu Chen

**Affiliations:** ^1^ Department of Orthopedics, Kaohsiung Veterans General Hospital, Kaohsiung, Taiwan; ^2^ Division of Nephrology, Department of Internal Medicine, Kaohsiung Veterans General Hospital, Kaohsiung, Taiwan; ^3^ Department of Medical Education and Research, Kaohsiung Veterans General Hospital, Kaohsiung, Taiwan; ^4^ Department of Pharmacy, School of Pharmacy, Kaohsiung Medical University, Kaohsiung, Taiwan; ^5^ Department of Occupational Therapy, Shu-Zen Junior College of Medicine and Management, Kaohsiung, Taiwan; ^6^ Department of Biomedical Engineering, I-Shou University, Kaohsiung, Taiwan

**Keywords:** wide-awake local anesthesia no tourniquet, WALANT, general anesthesia, GA, distal radius, fracture, fixation

## Abstract

**Background:**

Distal radius fractures are treated using open reduction and internal fixation and using general anesthesia (GA) or regional blocks. A new technique, wide-awake local anesthesia with no tourniquet (WALANT), allows this operation to be conducted in nonsedated patients without the use of tourniquets.

**Objective:**

We analyzed whether WALANT yields better outcomes than GA in the treatment of patients with distal radius fractures.

**Evidence Review:**

We searched the PubMed, Cochrane Library, Embase, and Scopus databases for cases of distal radius fractures treated using WALANT or GA. The outcomes of interest were duration of preparation for surgery, duration of surgery, blood loss, and length of postoperative hospitalization; visual analog scale (VAS), Mayo wrist score, and Quick Disabilities of the Arm, Shoulder and Hand (QuickDASH) questionnaire score on postoperative day 1; range of motion (ROM); time until bone union; and complication rate.

**Findings:**

We systematically reviewed 4 studies with a total of 263 patients (128 with WALANT and 135 with GA). In comparison with GA, WALANT required less time for preparation for surgery, shorter postoperative hospitalization, and lower postoperative day 1 VAS scores; however, blood loss was greater. Functional outcomes (ROM, QuickDASH score, and Mayo wrist score), complication rates, and times until union did not differ considerably between the two methods.

**Conclusion:**

The included studies demonstrated that durations of preparation for surgery and postoperative hospitalization were shorter and pain on postoperative day 1 was less severe with WALANT than with GA. Although blood loss in surgery was greater with WALANT, this technique is a novel and promising alternative to GA.

## Highlights

- A meta-analysis and systematic review compared wide-awake local anesthesia with no tourniquet (WALANT) versus general anesthesia (GA) in patients with a distal radius fracture.
- WALANT is a promising alternative to GA for its reliable, cost-effective, and time-saving qualities, especially in older patients and those with various comorbidities.
- Durations of preparation for surgery and postoperative hospitalization were shorter and pain on postoperative day 1 was less severe with WALANT than with GA.

## Introduction

Distal radius fracture, a common upper-limb injury worldwide, has a bimodal age distribution pattern: It tends to occur in young adults with high-energy trauma and in older adults with osteoporosis (1). Open reduction and internal ﬁxation provide immediate stability to support patients’ quick return to daily life and regular work. Such fixation with plates can be performed by administering regional blocks (e.g., Bier’s block and brachial plexus block) or general anesthesia (GA) to patients. With both types of anesthesia, a tourniquet can be used to control bleeding and provide a clear surgical field. However, administering regional blocks is technically demanding and requires special equipment, and GA can be dangerous in patients at high risk for complications (2).

A new method of administering anesthesia, wide-awake local anesthesia with no tourniquet (WALANT), was conceived by Lalonde et al. (3) The WALANT technique involves the local administration of lidocaine and epinephrine into the surgical ﬁeld, thereby allowing to conduct the operation without using sedation or tourniquets (4). It is used mainly in numerous hand and wrist procedures such as trigger finger (5), carpal tunnel release (6), wrist arthroscopy for triangular fibrocartilage complex repair (7), radial forearm perforator flap (8), tendon repair or transfer (9, 10), and internal fixation or implant removal for metacarpal fractures (11).

In comparison with GA, WALANT can be performed in older patients and in those with various comorbidities. Intraoperative anesthetic monitoring is not required, and hand or wrist function can be assessed in real time with the cooperation of nonsedated patients (12). Furthermore, it precludes not only postoperative pain and swelling of soft tissues caused by the tourniquet (13) but also anesthetic care after surgery and complications such as nausea and vomiting caused by GA. Therefore, WALANT is more cost-effective because postoperative hospitalization is reduced (14) and the services of anesthesiologists and preoperative testing for sedation are not required (15).

Fixation of distal radius fractures necessitates a wide surgical field and complicated bony procedures. Therefore, our concern is whether WALANT is a feasible alternative to GA in surgical treatment. To compare the effectiveness of WALANT with that of GA in surgical treatment, we conducted a systematic review and meta-analysis. The outcomes of interest were duration of preparation for surgery, duration of surgery, blood loss, and duration of postoperative hospitalization; visual analog scale (VAS) score, Mayo wrist score, and Quick Disabilities of the Arm, Shoulder and Hand (QuickDASH) questionnaire score on postoperative day 1; range of motion (ROM), measured by wrist flexion and wrist extension; time until bone union; and complication rate.

## Methods

### Search Strategy

The study was conducted according to the Preferred Reporting Items for Systematic Review and Meta-Analysis guidelines. We searched the PubMed, the Cochrane Library, Embase, and Scopus databases for publications up to February 10, 2021, with the following search terms: “distal radius fracture,” “wide-awake local anesthesia no tourniquet,” and “WALANT” (search details are listed in the supplement). Randomized controlled trials (RCTs) and cohort studies were included, as were studies in which distal radius fractures were treated using a surgical intervention and WALANT or GA. Perioperative variables and clinical outcomes were included. Comments, letters, case reports, case series, editorials, proceedings, and personal communications were excluded. The search strategies are illustrated in the supplement. We also manually searched the reference lists of the relevant studies to retrieve additional studies. No language or date restriction was applied in this systematic search.

Because a meta-analysis does not involve human subjects, institutional review board review, ethical approval, and informed consent were not required.

### Study Selection and Data Extraction

Studies were reviewed and selected by two independent reviewers. When study eligibility was uncertain, a third reviewer was consulted. The following information was extracted from the included studies: the name of the ﬁrst author, year of publication, study design, sample size, participants’ ages, follow-up period, distal radius fracture classification, WALANT solutions, injection procedure, postoperative medication regimen, and surgical outcomes.

### Quality Assessment

Two independent reviewers used the Newcastle–Ottawa Scale to assess the quality of the included retrospective cohort studies in terms of the selection bias (four items), comparability bias (one item), and outcome bias (three items). With the exception of one comparability item that was rated a maximum of two stars, each item was assigned one star at most if the quality of the study was high. The highest possible rating on the Newcastle–Ottawa Scale is 9 stars. A study with a score of 8 or 9 stars was recognized as high quality; 5–7 stars, as moderate or low quality; and fewer than 5 stars, as poor quality. After reviewers independently rated all studies, any disagreements were resolved through discussion.

RCTs were appraised by the Cochrane Collaboration’s Risk of Bias Tool (16) in the following six categories: (1) selection bias (use of random sequencing generation), (2) selection bias (use of allocation concealment), (3) detection bias (degree of “blinding” of outcome assessment), (4) performance bias (degree of “blinding” of participants and personnel), (5) attrition bias (presence of incomplete outcome data), and (6) reporting bias (as selective reporting). Each category was provided with one of three ratings: low, high, and unclear risk of bias. The overall risk of bias was considered low when all domains were rated as having a low risk of bias. It was considered unclear if at least one domain was considered to have an unclear risk of bias, but no domain was rated as having a high risk of bias; the overall risk of bias was otherwise considered high. After all studies were rated independently, any disagreements were resolved through discussion.

### Outcome Measures

Outcomes of interest were perioperative variables and clinical outcomes such as duration of preparation for surgery, duration of surgery, blood loss, and length of postoperative hospitalization; VAS score, Mayo wrist score, and QuickDASH score on postoperative day 1; ROM, measured by wrist flexion and wrist extension; time to bone union; and complication rate.

### Statistical Analysis

For the assessment of continuous data, we used mean differences (MDs) with corresponding 95% conﬁdence intervals (CIs). For the assessment of dichotomous data, we used relative risk and 95% CIs. A *p* value of <0.05 was considered statistically signiﬁcant. To assess the heterogeneity of the studies, we used Cochran’s Q test with the *Ι*^2^ statistic. The *Ι*^2^ values were deﬁned as follows: 0%–24% heterogeneity was considered low; 25%–49% heterogeneity was considered moderate; 50%–74% heterogeneity was considered high; and 75%–100% heterogeneity was considered extremely high. Because the number of studies included in the meta-analysis was small, heterogeneity tests had low statistical power (17), and because we observed heterogeneity between studies, random-effects models were conservatively applied for the meta-analysis (18). In addition, the National Research Council recommended the use of random-effects approaches for meta-analyses and the exploration of sources of variation in study results (19). Pooled effects sizes were calculated, and a two-sided *p* value of <0.05 was considered to indicate statistical signiﬁcance. We used the statistical software Comprehensive Meta-Analysis, version 3.0 (Biostat, Englewood, NJ, USA) to perform all analyses.

## Results

### Search Results

**Figure 1:**
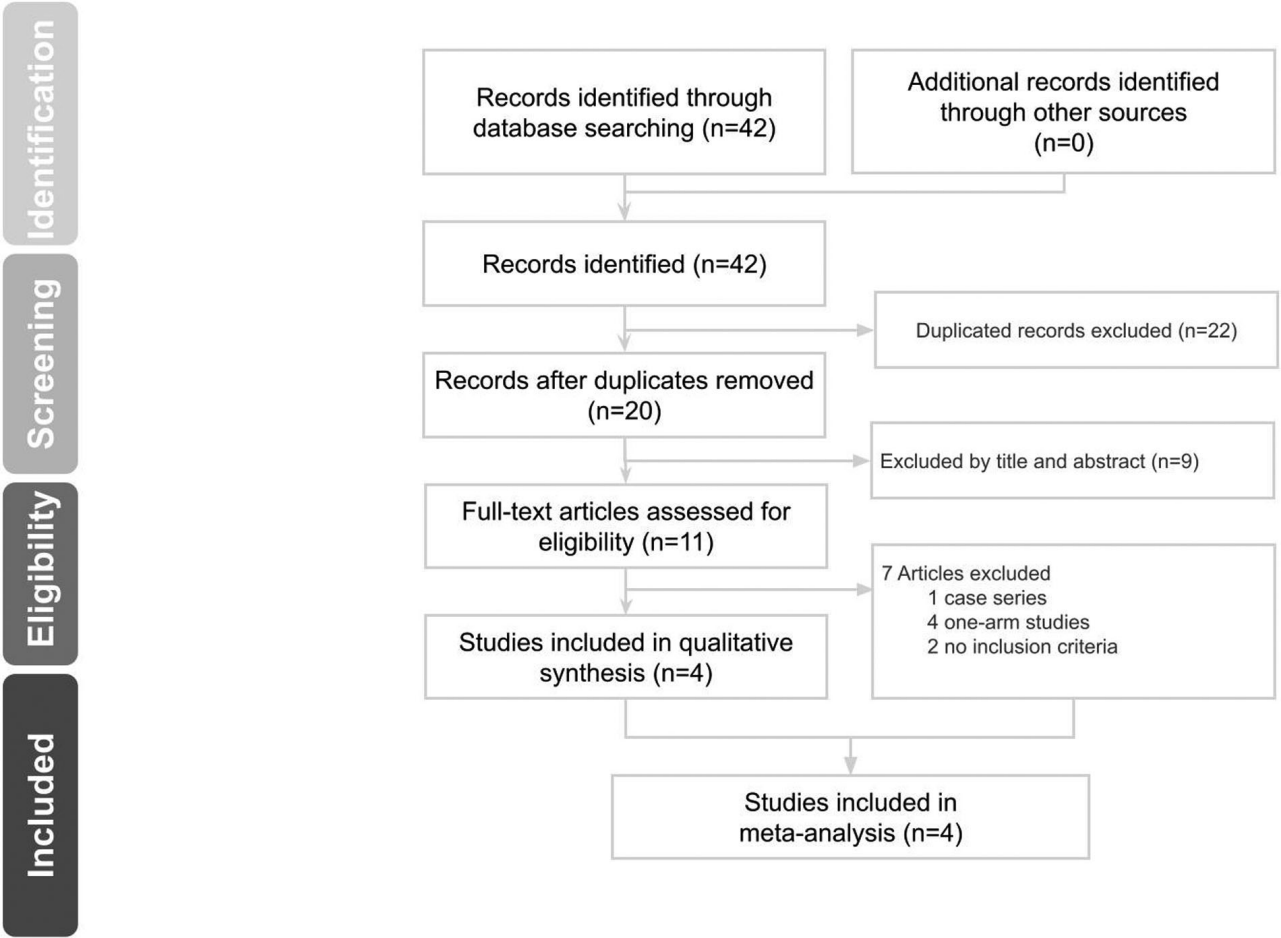
PRISMA ﬂow diagram of study selection.

We identified 42 potentially eligible studies in the initial search (Figure 1). We excluded 22 duplicates and 9 irrelevant studies by reviewing titles and abstracts. The remaining 11 studies underwent full-text review, and 7 were excluded because they were case series or one-arm studies or had different inclusion criteria. Four studies (2, 20–22) were thus included in the systematic review.

### Study Characteristics

Table 1 summarizes the main demographics of these four studies. Huang et al. (21) and Yi et al. (22) conducted retrospective cohort studies, and Tahir et al. (2) and Hamid et al. (20) conducted RCTs. The total number of patients in these studies was 263; 128 underwent surgery with WALANT, and 135 with GA. The Arbeitsgemeinschaft für Osteosynthesefragen (AO)/Orthopedic Trauma Association (OTA) classification varied across studies; with C2 fractures being the most common (22.1%), followed by A2 fractures (15.2%). The mean ages of patients among the studies ranged from 41 to 65 years. The lengths of follow-up ranged from 4 weeks to 1 year.

**Table 1:** Demographic characteristics of the studies included in the systematic review.

Study	Type	Intervention	Size (M/F)	Follow-up	Age (mean ± SD)	Fracture pattern (number of patients)	Inclusion criteria	Exclusion criteria	WALANT solution	Injection	Postoperative medication	Approach	Implant choice
Huang et al. (2019) (21)	RCS	WALANT	21 (8/13)	1 y	65.29 ± 15.47	A2 (3), A3 (6), B2 (5), C1 (3), C2 (4)	Acute traumatic injury, closed, unilateral distal radius fractures	Associated injury in other organs Multiple fractures Combined accessory wrist arthroscopic procedure	5–10 mL 1% lidocaine + 1:40,000 epinephrine	3–5 mL 1% lidocaine hematoma block → WALANT solution injected on volar side of DR → 5 mL WALANT solution beneath PQ	Tramadol, 37.5 mg/325 mg; acetaminophen twice/day	Henry	Volar locking Plate (2.4 mm LCP Distal Radius system, Synthes)
		GA	26 (9/17)		62.31 ± 14.42	A2 (9), A3 (5), B2 (2), C1 (1), C2 (8), C3 (1)			NA	230 mg Hg tourniquet			
Tahir et al. (2020) (2)	RCT	WALANT	55 (31/24)	2 and 6 wk, 1 y	46.6 ± 10.91	A2 (6), A3 (5), B1 (11), B3 (5), C1 (7), C2 (15), C3 (6)	Isolated closed fracture of the distal radius within 10 days	Open fracture of the distal radius Bilateral distal radius fracture Active infection in the body Contraindication for WALANT Noncooperative patients Bleeding tendency Hypersensitive to lidocaine Polytrauma patients	0.9% normal saline + 2% lidocaine + 1:100,000 epinephrine	3–5 mL 2% lidocaine hematoma block → WALANT solution injected on four skin points where 2 cm apart from the distal wrist crease → additional 5 mL WALANT solution beneath PQ	Tramadol, 37.5 mg/325 mg; acetaminophen, twice/day; calcium supplements	Henry	Volar Locking plates (Double medical Technologies, Fujian, China)
		GA	56 (28/28)		49.7 ± 9.3	A2 (5), A3 (7), B1 (6), B3 (9), C1 (7), C2 (9), C3 (13)			NA	250 mg Hg tourniquet			
Yi et al. (2020) (22)	RCS	WALANT	20 (19/1)	4 wk	41.7 ± 16.37	A2 (1), A3 (2), B1 (1), B2 (2), B3 (5), C1 (3), C2 (4), C3 (2)	Distal radius fracture Patients consent to participate	NA	50 mL 0.9% normal saline + 50 mL lidocaine HCl 2% + 1 mL adrenaline acid tartrate 0.18% 1 mg/mL + 10 mL sodium bicarbonate 8.4%	WALANT solution injected subcutaneously along the modiﬁed Henry skin incision, 1 cm beyond the incision site and three dots^a^ along radial border of radius	NA	Modified Henry	Volar Plating
		GA	20 (10/10)		44.3 ± 17.63	A2 (1), A3 (4), B1 (2), B2 (3), B3 (3), C1 (2), C2 (3), C3 (2)			NA	NA			
Hamid et al. (2021) (20)	RCT	WALANT	32 (21/11)	3 and 6 wk, 3 and 6 mo	47.19 ± 8.19	A2 (9), A3 (2), B1 (2), B2 (2), B3 (6), C1 (3), C2 (6), C3 (2)	Distal radius fracture Patients consent to participate	Peripheral vascular disease Diabetes mellitus Ischemic heart disease Psychiatric illness Allergy to lignocaine Patient demanding GA	50 mL of normal saline + 50 mL of lidocaine 2% +1 mL of 1:1,000 adrenaline solution + 10 mL of 8.4% sodium bicarbonate	10 mL of WALANT solution injected subcutaneously along the modified Henry skin incision, 30 mL was injected into the periosteal layer at three dots^a^	Tramadol, 50 mg three times a day; paracetamol, 1,000 mg four times a day	Modified Henry	Volar Plating
		GA	33		49.48 ± 6.02	A2 (6), A3 (2), B1 (3), B2 (6), B3 (2), C1 (2), C2 (9), C3 (3)			NA	NA			

*WALANT, wide-awake local anesthesia and no tourniquet; RCS, Retrospective cohort study; DR, distal radius; PQ, pronator quadratus; GA, general anesthesia; RCT, Randomized controlled trial; NA, not applicable.*

^a^ *At each dot, 10 mL of the WALANT solution was injected at different angles into the volar (4 mL), lateral (2 mL) and posterior (4 mL) aspects of the radius within the periosteal layer.*

### Summary of Outcomes

Table 2 lists the outcomes of interest in each study. Preparation time for surgery was considerably shorter with WALANT than with GA in the studies conducted by Tahir et al. (2) and Huang et al. (21); duration of surgery was considerably longer with WALANT in Hamid et al.’s study (20) but substantially shorter in Tahir et al.’s study (2) Blood loss was considerably greater with WALANT in the studies conducted by Tahir et al. (2) and Huang et al. (21) Duration of hospitalization was substantially shorter after surgery with WALANT in the studies conducted by Tahir et al. (2), Huang et al. (21), and Yi et al. (22) VAS scores were considerably lower 1 day after surgery with WALANT in the studies conducted by Tahir et al. (2) and Huang et al. (21); the QuickDASH score was substantially lower (which indicates better functional outcome) after surgery with WALANT in Hamid et al.’s study (20); in all these studies, Mayo wrist scores, wrist flexion, wrist extension, time to bone union, and complication rates did not differ between patients who underwent surgery using WALANT and surgery using GA. The total complication rate was 0.8% after surgery using WALANT (1 of 128 patients) and 5.9% after surgery using GA (8 of 135 patients). The proportions of patients requiring revision surgery were 0.8% after initial surgery with WALANT (1 of 128 patients) and 1.5% after surgery with GA (2 of 135 patients).

**Table 2:** Patients’ outcomes presented in the studies.

Study	Intervention	Size	Preparation time, min (mean ± SD)	Surgery time, min (mean ± SD)	Blood loss, mL (mean ± SD)	Post-operation Day 1 VAS (mean ± SD)	Postoperative hospital stay, days (mean ± SD)	Union time, wk (mean ± SD)	Extension (mean ± SD)	Flexion (mean ± SD)	Mayo wrist score (mean ± SD)	QuickDASH score (mean ± SD)	Complication (case / total)	Revision (*n*)
Huang et al. (2019) (21)	WALANT	21	25.38 ± 4.59	68.10 ± 9.28	22.62 ± 6.82	1.95 ± 0.67	1.38 ± 0.5	20.76 ± 4.35	50.24 ± 9.28	67.14 ± 9.95	86.67 ± 7.13	NA	0 / 21	0
	GA	26	37.31 ± 11.16	64.42 ± 10.42	8.62 ± 9.23	3.27 ± 1.28	2.46 ± 0.71	22.46 ± 4.17	49.42 ± 6.22	71.35 ± 8.19	84.04 ± 7.35		0 / 26	0
Tahir et al. (2020) (2)	WALANT	55	23.0 ± 3.85	61.30 ± 9.28	23.40 ± 8.50	1.20 ± 0.62	0.20 ± 0.50	15.3 ± 2.31	54.8 ± 6.45	65.9 ± 6.01	86.3 ± 5.08	10.2 ± 2.80	0 / 55	0
	GA	56	33.7 ± 5.81	68.80 ± 14.97	11.50 ± 4.25	3.00 ± 1.24	1.20 ± 0.78	15.8 ± 2.54	52.9 ± 4.45	64.3 ± 4.47	87.3 ± 5.13	10.2 ± 2.99	3 / 56	0
Yi et al. (2020) (22)	WALANT	20	NA	86 (*p* = 0.079)	49 (*p* = 0.67)	NA	1 (*p* = 0.009)	NA	NA	NA	NA	NA	1 / 20	1
	GA	20		102	63		2						3 / 20	0
Hamid et al. (2021) (20)	WALANT	32	NA	61.22 ± 7.72	14.88 ± 5.29	2.66 ± 0.60	NA	NA	70.47 ± 8.17	72.81 ± 7.18	NA	4.09 ± 0.89	0 / 32	0
	GA	33		55.30 ± 7.90	13.03 ± 2.78	2.85 ± 0.80			71.36 ± 5.63	74.55 ± 5.64		4.45 ± 0.87	2 / 33	2

*SD, standard deviation; VAS, visual analog scale; QuickDASH, Quick Disabilities of the Arm, Shoulder and Hand questionnaire; WALANT, wide-awake local anesthesia and no tourniquet; GA, general anesthesia; NA, not applicable.*

### Meta-Analysis

All studies were included in the meta-analysis and evaluated for differences in the outcomes of interest (Figure 2). Preparation for surgery using WALANT was significantly shorter than that for surgery using GA (MD = −10.841; 95% CI, −12.570 to −9.113; *p* = 0.000), with no heterogeneity observed in these two studies (2, 20) (*Ι*^2^ = 0%; *χ*^2^ = 0.198; *p* = 0.656; Figure 2A). Duration of surgery did not differ significantly between the two techniques (MD = −1.511; 95% CI, −9.798 to 6.777; *p* = 0.721), and extreme heterogeneity was found among the four studies (*Ι*^2^* *= 87.40%; *χ*^2^ = 23.822; *p* = 0.000; Figure 2B). Blood loss was significantly greater during surgery using WALANT than during surgery using GA (MD = 8.755; 95% CI, 0.909–16.602; *p* = 0.029), and extreme heterogeneity was noted among the four studies (*Ι*^2^ = 93.725%; *χ*^2^ = 47.810; *p* = 0.000; Figure 2C). Hospitalization was significantly shorter after surgery using WALANT than after surgery using GA (MD = −1.023; 95% CI, −1.218 to −0.829; *p* = 0.000), and no heterogeneity was noted among three studies (2,21,22) (*Ι*^2^ = 0%; *χ*^2^ = 0.135; *p* = 0.935; Figure 2D). VAS scores were significantly lower the day after surgery using WALANT (MD = −1.097; 95% CI, −2.192 to −0.003; *p* = 0.049), and extreme heterogeneity was observed among three studies (2, 21, 22) (*Ι*^2^* *= 95.083%; *χ*^2^ = 40.674; *p* = 0.000; Figure 2E). No significant differences between the two techniques were found for the following outcomes of interest: Mayo wrist scores (MD = 0.321; 95% CI, −3.102 to 3.743; *p* = 0.854), for which heterogeneity between two studies (2, 21) was high (*Ι*^2^* *= 58.505%; *χ*^2^ = 2.410; *p* = 0.121; Figure 2F); QuickDASH scores (MD = −1.234; 95% CI, −3.544 to 1.076; *p* = 0.295), for which heterogeneity between two studies (2, 21) was extremely high (*Ι*^2^ = 93.71; *χ*^2^ = 15.904; *p* = 0.000; Figure 2G); wrist flexion (MD = −0.856; 95% CI, −4.126 to 2.414; *p* = 0.608), for which heterogeneity among three studies (2, 21, 22) was high (*Ι*^2^ = 67.724%; *χ*^2^ = 6.197; *p* = 0.045; Figure 2H); wrist extension (MD = 1.107; 95% CI, −0.530 to 2.745; *p* = 0.185), for which no heterogeneity was found among three studies (2, 21, 22) (*Ι*^2^ = 0%; *χ*^2^ = 1.910; *p* = 0.385; Figure 2I); time until bone union (MD = −0.644; 95% CI, −1.492 to 0.203; *p* = 0.136), for which no heterogeneity was found between two studies (2, 22) (*Ι*^2^ = 0%; *χ*^2^ = 0.814; *p* = 0.367; Figure 2J); and complication rates (relative risk = 0.294; 95% CI, 0.072–1.204; *p* = 0.089), for which no heterogeneity was found among four studies (2, 21, 22) (*Ι*^2^ = 0%; *χ*^2^ = 0.822; *p* = 0.844; Figure 2K).

**Figure 2:**
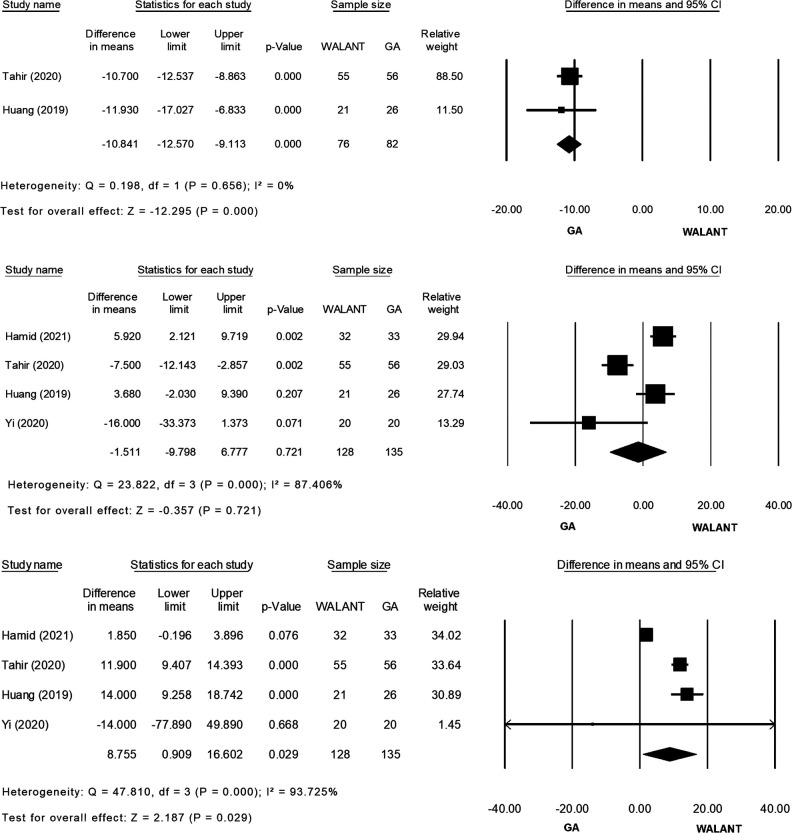
(Continued)

**Figure 2:**
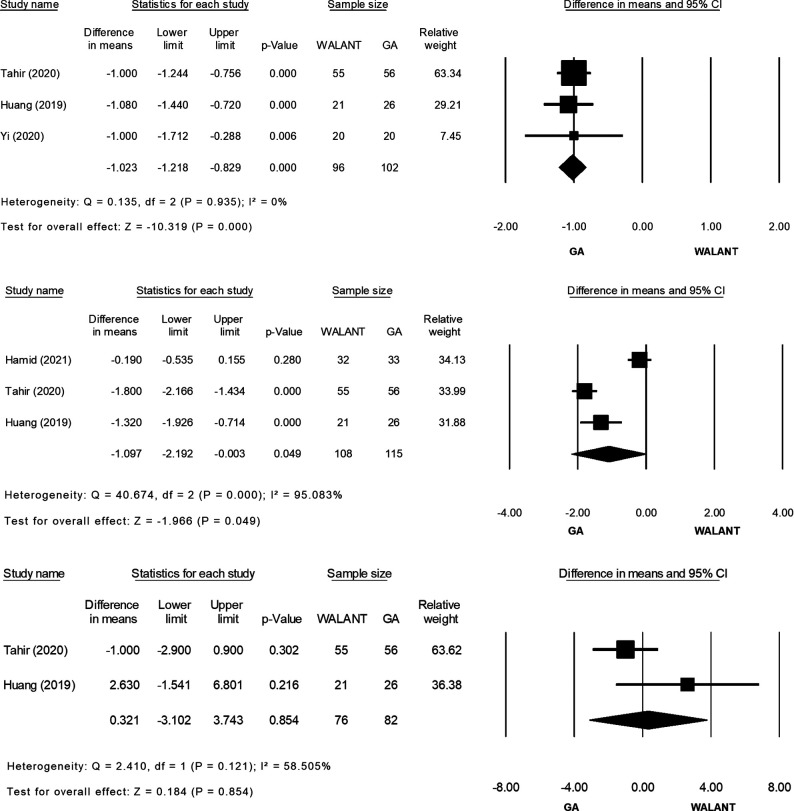
(Continued)

**Figure 2:**
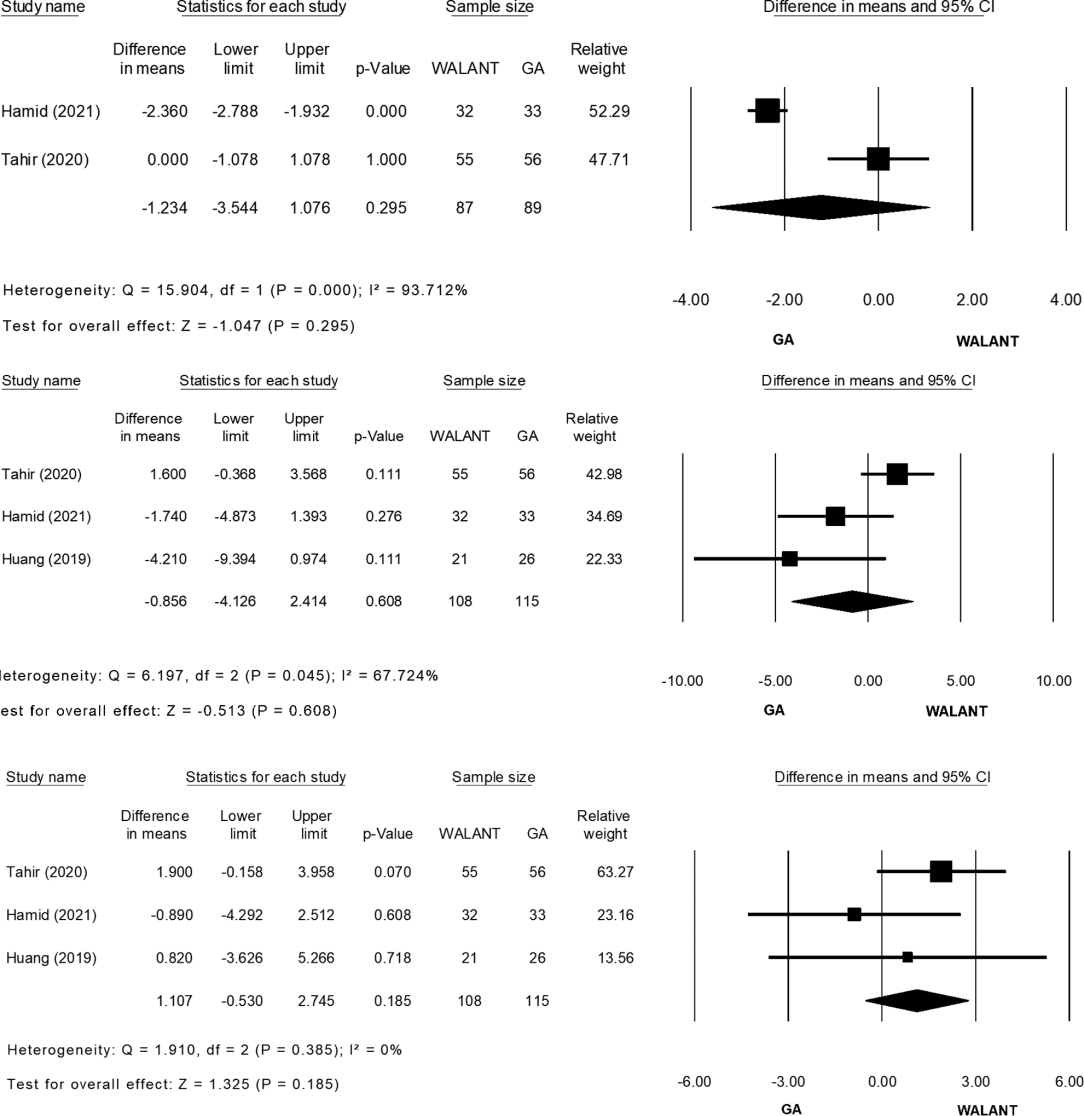
(Continued)

**Figure 2:**
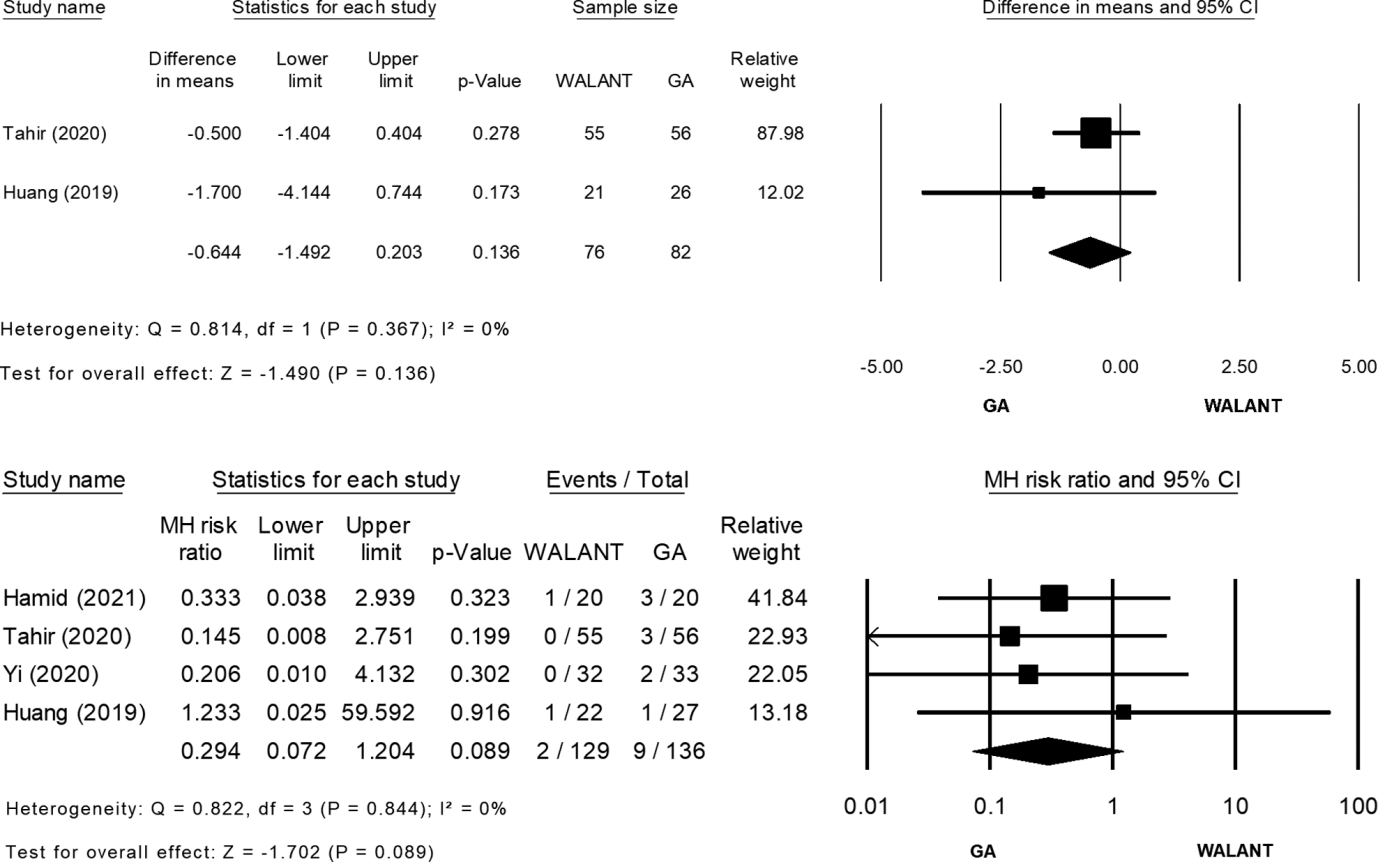
(**A**) Forest plot of Preparation time for surgery (minute) comparing WALANT with GA. (**B**) Forest plot of Surgery time (minute) comparing WALANT with GA. (**C**) Forest plot of Blood loss (mL) comparing WALANT with GA. (**D**) Forest plot of Postoperative Hospital stays comparing WALANT with GA. (**E**) Forest plot of Post-operation day 1 visual analog scale (VAS) comparing WALANT with GA. (**F**) Forest plot of Mayo Wrist score comparing WALANT with GA. (**G**) Forest plot of Quick DASH score comparing WALANT with GA. (**H**) Forest plot of Wrist Flexion (degree) comparing WALANT with GA. (**I**) Forest plot of Wrist Extension (degree) comparing WALANT with GA. (**J**) Forest plot of Union time (week) comparing WALANT with GA. (**K**) Forest plot of Complication rate comparing WALANT with GA.

### Quality Assessment

The Newcastle–Ottawa Scale was used to evaluate the quality of the studies by Huang et al. (21) and Yi et al. (22) (Table 3), and the Cochrane Collaboration’s Risk of Bias Tool was used to evaluate bias in the studies conducted by Tahir et al. (2) and Hamid et al. (20) The studies conducted by Huang et al. (21) and Yi et al. (22) were considered to have superior quality. The risk of bias in the studies conducted by Tahir et al. (2) and Hamid et al. (20) was considered high caused by performance bias (“blinding” of participants and personnel in both studies).

**Table 3:** Risk of bias assessment of included studies.

Study	Newcastle–Ottawa scale	Selection bias				Comparability bias	Outcome bias			Quality
		Representativeness of the exposed cohort	Selection of the non-exposed cohort	Ascertainment of exposure	Outcome of interest was not present at start of study	Comparability of cohorts on the design	Assessment of outcome	Follow-up long enough for outcomes to occur	Adequacy of follow-up of cohorts	
Huang et al. (2019) (21)	RCS	*	*	*	*	*	*	*	*	High
Yi et al. (2020) (22)	RCS	*	*	*	*	*	*	*	*	High
**Study**	**ROB**	**Selection bias**			**Reporting bias**	**Performance bias**	**Detection bias**	**Attrition bias**	**Other bias**	**Risk of bias**
		**Random sequence generation**	**Allocation concealment**							
Tahir et al. (2020) (2)	RCT	Low	Low		Low	High	Low	Low	Low	High
Hamid et al. (2021) (20)	RCT	Low	Unclear		Unclear	High	Unclear	Low	Low	High

On Newcastle–Ottawa scale, high quality: 8–9 stars (*), moderate quality: 5–7 stars (*), low quality: <5 stars (*).

*RCS, Retrospective cohort study; RCT, Randomized controlled trial; ROB, The Cochrane Collaboration’s Risk of Bias Tool.*

## Discussion

For a long time, epinephrine was considered the leading cause of severe complications, such as gangrene or necrosis, following hand or wrist surgeries. However, Lalonde et al. showed that epinephrine injections produced no digital tissue loss or skin necrosis in 3,110 consecutive patients (3). Because epinephrine was proven safe, McKee et al. (23, 24) proposed waiting approximately 30 min after administering the injection to maximize hemostasis before making an incision. The widely accepted maximal dose of lidocaine that is believed to be safe for upper extremity surgery is 7 mg/kg (25). The addition of epinephrine prolongs the duration of action of lidocaine from 30–60 min to 120–360 min (26). The acidity of the solution (pH: 4.2) is likely to cause pain to the patient during injection; therefore, buffering the WALANT solution (1% lidocaine with 1:100,000 epinephrine) with 8.4% sodium bicarbonate at a ratio of 1:20 to reach the physiologic pH of 7.4 was recommended (27, 28). According to a Cochrane review (29), patients favor buffered lidocaine over unbuffered lidocaine.

WALANT appears to be a safe, cost-effective, and time-efficient technique. Compared with GA, the primary advantages of WALANT are that (1) because a tourniquet is not required, postoperative discomfort, muscle necrosis, and nerve injury can be avoided. (2) Deep sedation is not essential; thus, recovery is faster, fewer side effects such as nausea and vomiting occur, and the anesthesia risk in older patients with multiple comorbidities is lower. (3) Patients do not need to fast overnight, which minimizes the risk of glycemic change in patients with diabetes before surgery. (4) Preoperative testing—such as blood tests, chest radiographs, electrocardiography, and medical clearance—and the services of an anesthesiologist and postoperative anesthetic care are not required, which thereby saves time and decreases costs (30). However, WALANT application was restricted in case of patients with needle phobia, peripheral vascular diseases or active infection, bleeding tendency, abnormal clotting profile, and hypersensitivity to lidocaine (2).

A mean saving of USD 1320 in health care costs, including anesthesia cost, preoperative cost, and postoperative cost, was noted in an American study in which the cost of WALANT was compared with that of GA in surgery for carpal tunnel syndrome (31). Furthermore, despite the MDs in annual household incomes between the United States and Pakistan, Tahir et al. demonstrated that WALANT costs less in hospital spending (USD 202.10; *p* < 0.001) compared with GA in terms of repairs of distal radius fracture in both countries (2). In contrast, Caggiano et al. (32) showed that the choice of anesthesia considerably affected total nonsurgical time, room turnover time, in-room presurgical time, and in-room postsurgical time; for instance, local anesthesia reduced the total nonsurgical time by 40% in comparison with GA. In the repair of distal radius fractures, WALANT, a safe, cost-effective, and time-efficient technique, resulted in better patient satisfaction (*p* < 0.001) compared with GA (2).

In this quantitative, comparative meta-analysis of four studies (two retrospective cohort studies and two RCTs) with a total of 263 patients, C2 and A2 were the most common types of distal radius fractures according to the AO/OTA classification. With WALANT, preparation for surgery and postoperative hospitalization were shorter and VAS scores on postoperative day 1 were lower, but blood loss was greater in comparison with GA. For conventional GA, an anesthesiologist and nursing staff must be available, in addition to information about the patient’s medical history and vital sign monitoring equipment, before and after anesthesia induction, and the patient needs to be intubated. WALANT does not require the abovementioned staff and procedures, and surgical preparation time can thus be substantially shortened. Because a tourniquet is not used, the pain in the upper arm is reduced, and the painful swelling and ecchymosis caused by the immediate venous return after release of the tourniquet can be avoided. When pain decreases and the effects of deep sedation, including nausea, vomiting, and dizziness, do not occur, hospitalization is considerably shortened. However, no significant differences were found between the two techniques in terms of the functional outcome (ROM, QuickDASH score, and Mayo wrist score), complication rate, and time to bone union. The total complication rates were lower after surgery using WALANT (0.8%) than after surgery using GA (5.9%), and the proportions of patients requiring revision surgery using WALANT (0.8%) were lower than those using GA (1.5%).

Complications with GA occurred in three patients in the study by Yi et al. (20), who experienced nausea and vomiting for 1 day; in one patient with attrition injury and two patients with mild wound inflammation in the study by Tahir et al. (2); and in one patient with screw penetration of the wrist joint and another with distal radioulnar joint dissociation (both of whom underwent subsequent revision surgery) in the study by Hamid et al. (20). Conversely, complications with WALANT—a reduction in radial inclination and an increase in dorsal tilt, treated using revision surgery with K-wire augmentation—were noted in one patient in the study by Yi et al.. The definitive functional outcome and complication rate after surgery should depend on the severity of the fracture in terms of whether it involves the joint surface, the appropriate selection and placement of an implant, and the reduction and surgical technique. In addition to disparate surgery-related problems, different postoperative rehabilitation protocols and medications may have contributed to the discrepancies in functional outcomes among the studies. Therefore, the present study of these two different anesthesia approaches revealed only differences in immediate recovery and pain after surgery but not in different functional outcomes and complication rates.

This study had several limitations. First, the follow-up periods in most studies varied and were no more than 1 year; several outcomes and complications may have emerged during a more extended follow-up period. Second, these four studies with small sample sizes (two nonrandomized controlled studies and two randomized controlled studies with an overall high risk of bias) may have been affected by various biases and a low statistical power. Third, the high heterogeneity among studies that was observed for some outcomes may be attributable to different covariates. Incongruous distribution among different types of distal radius fractures and different WALANT solution formulas presumably resulted in heterogeneity in surgery duration and blood loss among studies. Last, despite the apparently lower complication rate using WALANT compared with GA, some of the complications that necessitated secondary revisions were associated with technical problems rather than problems associated with anesthesia. The misattribution of these complications might cause misclassification bias. There are still some distinctions in the anesthesia methods used in these included articles. Moreover, different epinephrine and xylocaine concentrations were administered. Further research is essential for further investigation and objective observations of factors such as oozing status, difficulty of reduction, patients’ pain perceptions, and variation in vital signs during surgery.

In summary, this systemic review and meta-analysis demonstrated that WALANT, in comparison with GA, shortened the time required for the preparation of surgery, shortened postoperative hospitalization, and resulted in less pain 1 day after distal radius fracture fixation; however, blood loss was greater with WALANT, which is a drawback. According to some studies (2, 21), even if blood loss is greater without the use of a tourniquet, it is still minimal and does not affect the operation. Otherwise, the final functional outcome and complication rates did differ considerably between the two different anesthetic approaches. Although this study showed that WALANT is a favorable alternative to GA in terms of reliability, cost-effectiveness, and time saved, each patient should be treated on a case-by-case basis because both treatments have benefits and drawbacks.

## Data Availability

The original contributions presented in the study are included in the article/Supplementary Material, further inquiries can be directed to the corresponding author/s.
